# Supplementary material for: Development of a Pandemic Resilience Competence Model for Healthcare Professionals—Individual and Organisational Aspects
Source: Int J Environ Res Public Health. 2025 May 1;22(5):712. doi: 10.3390/ijerph22050712 (PMC12110828; doi:10.3390/ijerph22050712)
Supplement: Supplementary file 1 [file ijerph-22-00712-s001.zip › ijerph-3489902-supplementary.pdf]

Supplementary Material 1

Table S1. Demographics for Austria

Number	Age	Gender	Job Description
1	28	F	Resident Gyn/OB
2	28	M	Nurse
3	27	F	Physiotherapist
4	36	M	Nursing Director
5	25	F	Dietician
6	29	F	Resident
7	73	M	Emergency Doctor
8	38	F	Nurse
9	27	M	Nursing assistant
10	41	M	Nursing Home Director

Table S2. Demographics for Portugal

Number	Age	Gender	Job Description
1	56	F	Medical Doctor
2	30	F	Occupational therapist
3	62	M	Radiology Technician
4	33	M	Nurse
5	53	F	Nurse-Chief
6	48	F	Medical Doctor
7	60	F	Physiotherapist
8	34	F	Occupational therapist
9	29	F	Nurse
10	40	F	Member of the Board of Directors General Hospital

Table S3.Demographics for UK

Number	Age	Gender	Job Description
1	-	M	Team Leader Care Home
2	-	M	Healthcare Assistant
3	-	F	Nurse
4	-	F	Nurse – Ward Manager
5	-	F	Healthcare Assistant
6	-	M	Senior Healthcare Assistant
7	-	F	Operations Support Manager
8	-	M	Senior Care Assistant
9	-	F	Senior Healthcare Assistant

10	-	F	Nurse - Team Leader Hospital

Table S4. Demographics for Germany

Number	Age	Gender	Job description
1	31	F	Health administration worker
2	55	F	Nurse
3	58	F	Nursing Director
4	46	F	Health administration worker
5	-	F	Physiotherapist
6	-	F	Nurse
7	-	-	Nurse
8	-	M	Physiotherapist
9	-	M	Physiotherapist
10	-	M	Health Technology Specialist

Table S5. Demographics for Italy

Number	Age	Gender	Job description
1	49	M	Specialist in Anesthesiology and Intensive Care Medicine
2	53	F	Head of Nursing Services
3	38	F	Social Worker
4	58	M	Jurist/ Lawyer
5	60	M	District Health Coordinator and Deputy Health Director
6	61	M	Primary Doctor
7	39	F	Specialist in Anesthesiology and Intensive Care Medicine
8	50	F	Nurse
9	64	M	Coordinating Head of Nursing Services and Deputy Director of Nursing
10	51	M	Responsible for Civil Protection and International Relations
